# Is there any intron sliding in mammals?

**DOI:** 10.1186/s12862-020-01726-0

**Published:** 2020-12-11

**Authors:** Irina V. Poverennaya, Nadezhda A. Potapova, Sergey A. Spirin

**Affiliations:** 1 Vavilov Institute of General Genetics, Russian Academy of Sciences, Moscow, Russia; 2 Institute of Mathematical Problems of Biology RAS-The Branch of Keldysh Institute of Applied Mathematics of Russian Academy of Sciences, Pushchino, Russia; 3 Institute for Information Transmission Problems (Kharkevich Institute) of the Russian Academy of Sciences, Moscow, Russia; 4 Faculty of Bioengineering and Bioinformatics, Lomonosov Moscow State University, Moscow, Russia; 5 Belozersky Institute of Physical and Chemical Biology, Lomonosov Moscow State University, Moscow, Russia; 6 Federal Science Center System Research Institute of the Russian Academy of Sciences, Moscow, Russia; 7 National Research University Higher School of Economics, Moscow, Russia

**Keywords:** Intron sliding, Introns, Gene annotation, Transcriptome, Intron evolution

## Abstract

**Background:**

Eukaryotic protein-coding genes consist of exons and introns. Exon–intron borders are conserved between species and thus their changes might be observed only on quite long evolutionary distances. One of the rarest types of change, in which intron relocates over a short distance, is called "intron sliding", but the reality of this event has been debated for a long time. The main idea of a search for intron sliding is to use the most accurate genome annotation and genome sequence, as well as high-quality transcriptome data. We applied them in a search for sliding introns in mammals in order to widen knowledge about the presence or absence of such phenomena in this group.

**Results:**

We didn’t find any significant evidence of intron sliding in the primate group (human, chimpanzee, rhesus macaque, crab-eating macaque, green monkey, marmoset). Only one possible intron sliding event supported by a set of high quality transcriptomes was observed between *EIF1AX* human and sheep gene orthologs. Also, we checked a list of previously observed intron sliding events in mammals and showed that most likely they are artifacts of genome annotations and are not shown in subsequent annotation versions as well as are not supported by transcriptomic data.

**Conclusions:**

We assume that intron sliding is indeed a very rare evolutionary event if it exists at all. Every case of intron sliding needs a lot of supportive data for detection and confirmation.

## Background

Eukaryotic genes consist of exons and introns whose borders, i.e. genomic coordinates, are evolutionarily conservative which means they are under the pressure of negative selection [1–4]. The changes of exon–intron boundaries might affect coding protein, therefore they are rare events and can be seen only on the long evolutionary distances [5]. Such changes differ from the alternative splicing, which is widespread in eukaryotes and does not affect exon–intron coordinates.

Various reasons for exon–intron boundaries alterations were observed. All of them happen as a result of mutations in splice sites or nearby, such as a birth or death of new splice sites resulted in a loss or acquisition of exons or their part [6–9]. But the rarest type of such alterations is called “intron sliding” (also known as “intron shifting”, “intron drift”, “intron slippage” or “intron migration”). Intron sliding is a movement of intron from one position to another within a gene [10]. In other words, during sliding the start and end of the certain intron move in the same direction for the same number of nucleotides. For example, if initially the intron start coordinate was 100 and the end coordinate was 500, after hypothetical intron sliding event coordinates would be 101 and 501, respectively. This means that coordinate changes for both sides are the same (in this case 1 bp shift). Potential mechanisms of intron sliding appearance were proposed [10–12] and it was shown that almost always they are no longer than 1 bp [12], but potentially might be of a length of 15 bp [13] or even 60 bp [14]. According to the different data [13, 15, 16] introns relocate their positions over the distances of 1–15 bp or even around 5% of the intron length. However, the longer the distance is, the more sceptical we might be about the presence of an intron sliding event rather than intron loss and gain in different positions.

As proper intron recognition and removal from transcripts require correct splice signals at the intron start and end, i.e. donor and acceptor splice sites, intron sliding might happen as a result of a series of mutations leading to creation of the alternative splice sites and loss of the old ones. It remains unclear whether canonical donor and acceptor splice sites GT-AG, which are the most common in eukaryotes, stay intact after intron sliding, or intron sliding rather happens in introns with non-canonical splice sites. The recently suggested molecular mechanism for 1 bp sliding [12] considers the importance of canonical splice sites and focuses on so-called stwintron (spliceosomal twin intron) that is an intron with nested internal introns that could be alternatively spliced.

Intron sliding does not affect the sequence of the transcript but can lead to intron phase changes [15], which might be important for alternative splicing. Let us consider a hypothetical example with the sequence at the exon–intron boundaries GAC|gtcct…tgag|CTAA where the splice site is shown by the vertical streak, donor and acceptor splice sites are underlined, intron’s sequence is highlighted in lowercase letters, while the sequences of neighbouring exons are in capital letters. If intron sliding, as it is generally predicted, happens as a result of the cut-and-paste mechanism then the sequence in the case of 1 bp sliding will look like this: GACC|gtcct…tgag|TAA. The intron sequence stays intact, while the exon sequences gain/lose 1 bp each at their border leading to intron phase change. However, if intron sliding happens in a series of point mutations at the exon–intron border or the presence of canonical splice signals stops being crucial for correct intron splicing for this particular case (according to [17], the number of non-canonical sites is probably greatly underestimated due to imperfect genome annotations), we might find the intermediate cases, e.g. GACG|tcct…tgaac|TAA (the point mutation G → A occurs at the end of intron leading to the creation of a new acceptor site AC| and shifting the exon–intron border). Here we might observe the changes not only in intron sequences but in the affecting codons as well.

Due to its ambiguous molecular mechanism of emergence, negative selection acting on splice sites and frequent errors in gene annotation, the existence of intron sliding is still debatable [10]. It seems that for the precise detection of such events the following is needed. First, we need the availability of well-assembled genomes or correct sequences of separate genes in case there is a search for intron sliding in a one certain gene. Second, essential are high-quality genome annotations and transcriptomes, to avoid annotation errors. In the very first intron sliding studies limited sets of data were used, e.g. only gene annotations and genomes, or even just genes [18, 19], and authors were cautious with claims about the existence of any intron sliding event. Later, a step with checking intron sliding using RNA-seq data was added, which helps to consider each potential case more accurately (e.g. see [20]). But yet authors of only a few studies are confident with the results of sliding intron presence [15, 16], while the majority of found cases failed after checking [21]. For instance, in [10] a list of 32 sliding events was examined and 20 cases were filtered out. However, the authors stayed skeptical about the remaining cases and suspected that most, if not all, of them were artifacts. In another study [22], 9 oomycete genomes were used and it was concluded that intron sliding is only accidental and plays a minor role in eukaryotic genome evolution [22]. Other works also showed that there is no intron sliding in *Cryptococcus* species [23] and in *Daphnia* [24].

Summarizing, there is a very small possibility for intron sliding occurrences. All of such events were observed using outdated data or genome annotations that had changed drastically in recent years. Some studies did not take into account transcriptomic data. Thus, each previously found possible case needs an accurate interpretation and verification.

We approached this task using the latest genome annotations, reference genome versions and high quality transcriptomic data for some mammalian species (including human, chimpanzee, rhesus macaque and mouse), for which the mentioned list of data was available. In order to understand the prevalence of intron sliding phenomena and, if it takes place, its characteristics, we identified and then verified each possible case of intron sliding between human and other mammals.

## Results

### Search for intron sliding events

Search for parallel shifts of exon–intron borders in the pairwise genome alignments between human and other species resulted in 35 potential intron sliding events. The maximum number of sliding hits (13) were found between human *Homo sapiens* and marmoset *Callithrix jacchus*. There were no identified cases between human and four other primates, such as chimp *Pan troglodytes*, rhesus macaque *Macaca mulatta*, crab-eating macaque *Macaca fascicularis* and olive baboon *Papio anubis*. All other genomes had 1–4 cases of potential intron sliding events relative to the human genome (Additional file 1: Table S1).

**Fig. 1:**
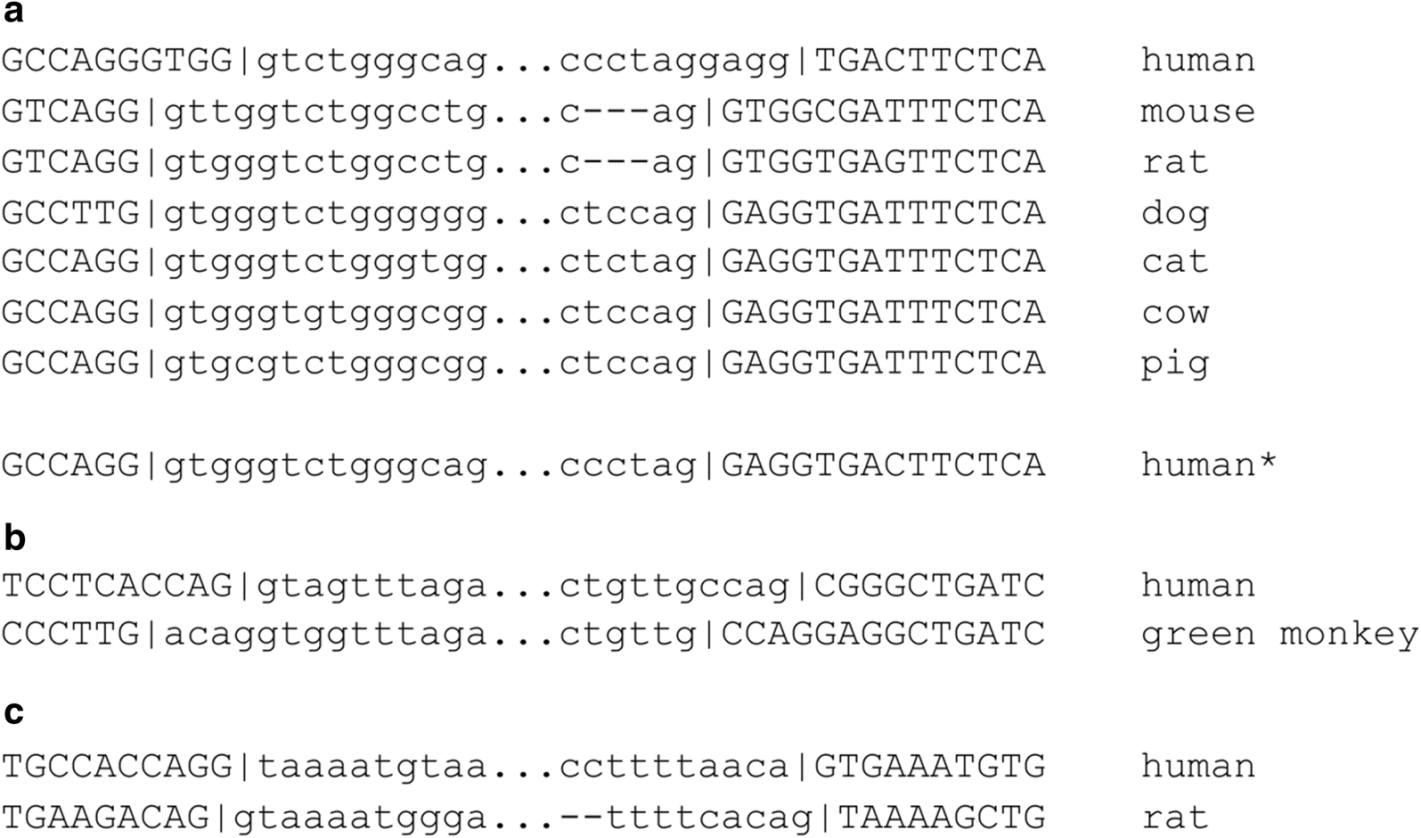
Possible changes of exon–intron boundaries over equal (**a**, **b**) and non-equal (**c**) distances found in genome pairwise alignments. **a** Intron sliding in SSPO homologous genes (Ensembl transcript ids human ENST00000378016, mouse ENSMUST00000169350, rat ENSRNOT00000035906, dog ENSCAFT00000007296, cat ENSFCAT00000024510, cow NM_174706, pig ENSSSCT00000045656; human* is an alternative SSPO transcript ENST00000475488 with unslided intron); **b** intron sliding in IGSF5 gene (human ENST00000380588, green monkey ENSCSAT00000006363.1); **c** intron border shifting over non-equal distances in DEFB133/Defb49 genes (human ENST00000398721, rat ENSRNOT00000060805)

Out of 35 detected cases only 30 were unique, as we found the same change of the exon–intron boundaries in human *SSPO* transcript (i.e. gene isoform) relative to its homologues in mouse *Mus musculus*, rat *Rattus norvegicus*, cat *Felis catus*, dog *Canis familiaris*, cow *Bos taurus*, and pig *Sus scrofa* (see Fig. 1a), suggesting that intron sliding happened in the human lineage. Among 30 cases the most frequent lengths of sliding were 2 bp and 4 bp (10 and 12 cases, respectively). The expected most frequent 1 bp intron sliding appeared to be only in five events (Additional file 1: Table S1). Canonical donor and acceptor splice sites GT-AG remained intact only in five sliding events in genes *SPIC*, *C3*, and *SWI5* (all in human/opossum), *CATSPER1* (human/marmoset) and *MICLK* (human/naked mole-rat). Two genes with detected sliding did not have canonical splice sites in the intron of interest: *IFT80* (human/naked mole-rat) and *PROX2* (homo/cow).

We found out that 5 of 30 human transcripts had low values of Transcript Support Level (TSL). TSL is a method to highlight the well-supported and poorly-supported transcript models for users in GENCODE [25]. Thus, the category TSL1 indicates that all exon–intron boundaries of this particular transcript are verified, TSL4, TSL5 and TSLNA mean that this transcript is predicted. For example, low TSL was observed for the transcript of *SSPO* gene where a sliding event was mentioned above. According to Ensembl [26], human *SSPO* is a transcribed unitary pseudogene, while in other organisms it is a protein-coding gene. It is commonly known that pseudogenes accumulate more mutations than normal genes [27], so it would be easy to expect that intron sliding occured in a pseudogene. However, the human transcript *SSPO-201* (Ensembl ID ENST00000378016), in which we predicted an intron sliding event, did not have the supporting transcriptomic evidence, meanwhile its alternative transcript *SSPO-205* (ENST00000475488) assigned with TSL1 had the same intron position as aligned *SSPO* mammalian homologues. Thus, this raises a big question about the presence of intron sliding here.

In addition, for the remaining 25 cases we revealed that some records of the UCSC ensGene annotation database are outdated relative to the current Ensembl release 99. Namely, 11 transcript records for our cases had been updated, with only one saving the same splice junction of interest (the pig transcript ENSSSCT00000012080 for *TMEM236* gene). The rest had changes in their exon–intron structure annotations leading to the elimination of predicted intron sliding events. Five more transcript records had been retired as they underwent serious sequence and annotation changes. Almost all the changes mentioned above were generally supported by mRNA and EST data from the UCSC Genome Browser, only in five cases there was no transcriptomic evidence to support any versions. Thus, we filtered out 15 cases from 25.

**Fig. 2:**
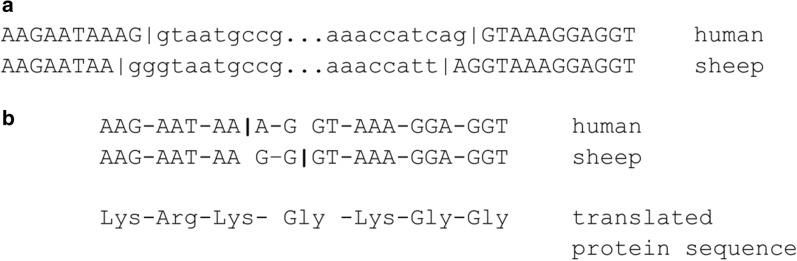
Intron sliding in EIF1AX gene between human and sheep (human transcript ENST00000379607.10 and sheep transcript NM_001145179). **a** Intron sliding alignment; **b** Exon nucleotide alignment with highlighted codons and translated protein sequence

Unfortunately, validation of the remaining ten annotated intron sliding cases was also complicated by the lack of transcriptomic data in the UCSC Genome Browser for some genes, e.g. for the genes of *H. glaber* (4 cases). That’s why we also could not verify the only one found case of putative intron sliding between human and another primate, green monkey *Chlorocebus sabaeus,* in the gene *IGSF5* (see Fig. 1b). The transcriptomic evidence for the transcripts of *PRPF6* (human/mouse), *TXLNB* (human/cow), *ZFYVE26* (human/pig) and *TMEM236* (human/pig) genes revealed the wrong annotation of the exon–intron boundaries in the current versions of the corresponding mouse’s, cow’s and pig’s transcripts and supported the intron positions such as in human, thus rejecting the predicted intron sliding. The only intron sliding event found to be supported by numerous mRNA and EST data was between human and sheep’s homologous genes *EIF1AX* (see Fig. 2). However, it should be noted that approximately the same proportion of transcriptomic data in the UCSC Genome Browser also supported the transcript variant without any sliding, thus, we regard this sliding event as dubious.

### Search for intron border shifting over non-equal distances

Though it is speculative to consider intron border shifts over non-equal distances as intron sliding, they could also affect intron positions and therefore gene structure evolution. We implemented the same pipeline as for traditional sliding events. As a result we found 22 cases where intron borders moved at non-equal distances (Additional file 1: Table S2). Among them only 18 cases appeared to be unique, because intron sliding in pig transcript ENSSSCT00000001516 (*POU5F1* gene) was found in five human transcripts that were in fact alternative (but matching) variants of the same gene on an alternative patches of human chromosome 6, and therefore represented only one unique case. No cases were found for chimp, crab-eating macaque, green monkey, naked mole-rat and opossum introns relative to human ones.

Change of intron boundaries could lead to frameshift errors, so the total length of shifts is expected to be multiple of three (and we found 9 such cases), otherwise compensatory mutations such as insertions or deletions would be necessary in order to keep frameshift intact (another 9 cases). For instance, there is a noted frameshift mutation in the current Ensembl annotation of the human transcript for mentioned above *POU5F1* gene. Also, only in four cases canonical dinucleotides GT-AG at the intron boundaries were not changed.

TSL assignment filtered out five cases of 18 with predicted human transcripts. Furthermore, according to the Ensembl Release 99, seven records of the analyzed mammalian transcripts were updated and four were retired due to considerable changes in the sequence and gene structure annotations. Six new annotations were supported by transcriptomic evidence from the UCSC Genome Browser, and it was difficult to verify others due to the insufficient transcriptomic data.

Thus, transcriptomic data mainly supported absence of exon–intron boundaries shifting, and we were able to identify only two cases with only one shifted border that might be annotated as alternative donor/acceptor splice sites, though presence of alternative splice sites at such close distance seems suspicious. Human transcript ENST00000421317 and marmoset transcript ENST00000421317 of *CUL2* gene as well as another human transcript ENST00000398402 with sheep transcript NM_001280713 of *CST1/CST3* genes shared their acceptor splice sites position, but had different donor splice sites. Both sites' positions changes were confirmed only in one intriguing case of *DEFB133/Defb49* human and rat genes. The only intron in the corresponding rat transcript ENSRNOT00000060805 changed its boundaries by 1 bp in both directions relative to the intron from human transcript ENST00000398721 (see Fig. 1c). The rat intron had canonical splice sites GT-AG, while the human intron had non-canonical dinucleotides TA-CA. Though being assigned with TSL1 the human intron had only two mRNA records (AY621330 and DQ012023) as transcriptomic evidence in the UCSC Genome Browser. Moreover, DQ012023 partially supported the same position of the acceptor splice site as a rat one had. In addition, verification of the rat transcript exon–intron structure was based on only one corresponding mRNA record presented in the UCSC Genome Browser.

## Discussion

Intron sliding is a rare evolutionary event when intron boundaries shift over a short equal distance. Due to its ambiguous molecular mechanism, frequent errors in gene annotation and quality of mapping exon–intron boundaries using transcriptomic data, the existence of intron sliding still has remained debatable.

In our study we compared exon–intron boundaries in the pairwise genome alignments for mammals from the UCSC Genome Browser and showed that sliding is indeed a very rare evolutionary phenomenon. The most complete genome annotations, many available transcriptomic and EST data for each species were used in order to avoid annotation errors resulting in invalid exon–intron coordinates. But even data of a high quality and accuracy, such as for the human and mouse, did not allow us to check the potential intron sliding in the two found cases between human and mouse genomes. We also did not find intron sliding in the primate group.

In general, we found 30 unique intron sliding cases, but after strict verification including transcriptomic and EST data support remained only one case of a length 2 bp between human and sheep in homologous genes *EIF1AX*. It occured in a sheep genome by using non-canonical rarely used splice sites GG-TT, which causes some distrust to the obtained result. Affected amino acid, lysine, remained intact in both cases, but codons were different, namely “AAA” and “AAG” (see Fig. 2). But even in this case approximately the same proportion of transcriptomic data supported the transcript variant without any sliding.

Other intron sliding cases were not supported, thus it is difficult to make a decision about the presence, or absence, of this sliding event in the analyzed group of species.

We also considered cases when intron boundaries changed on unequal distance. Very few examples of them were found but even they were poorly supported by transcriptomes and we stay sceptical about the existence of these borders’ changes.

While we found one speculative sliding event, the concept of intron sliding and possibility of its existence has been discussed for a long time in a number of previous studies [13, 14]. One of the main studies which asked and then confirmed its reality [13] was based on the analysis of intron positions in 40 conserved gene families. There were observed accurate intron sliding events of a length 1 bp, although such events were relatively rare and occured in < 5% of all introns, but it was proposed that they were very likely a real evolutionary phenomenon. In this study we received results that raised a question whether observed intron sliding (also of a length 1 bp as in previously mentioned study) is real in mammals or is it an artifact and non-existent event at all. In order to answer this question we attempted to reproduce the paper mentioned above [13]. Unfortunately, lack of detailed description of the used data complicated correct reconstruction and comparison of those results with the up-to-date data. However, we were able to revise three cases that were mentioned by authors as highly likely candidates of intron sliding events: in polyubiquitin genes of the alga *Volvox carteri* and the fungus *Schizophyllum commune*, in mammalian cholesterol esterase genes (from *Homo sapiens* and *Rattus norvegicus*) and in alcohol dehydrogenase II genes from two species of rice (*Oryza sativa* and *Oryza officinalis*). Current versions of sequences and exon–intron structure annotations (by the corresponded GenBank and Ensembl records) supported only one sliding event in polyubiquitin genes between *V. carteri* (GenBank accession number X74214) and *S. commune* (AF031628). For other mentioned cases sequence refinement and the following exon–intron border upgrade led to the elimination of potential sliding events between human and rat in *cel* gene (Ensembl transcript IDs ENST00000673714 and ENSRNOT00000014572, respectively) and between rice species in *adh2* gene (Ensembl transcript ID Os11t0210500-01 for *O. sativa* and GenBank accession number KP121892.1 for *O. officinalis*).

The main pitfall in intron sliding studies is the accuracy of genome annotations, which in turn depends on the quality of transcriptomes used for annotating. As we observed in our study, many annotations are outdated and are not in accordance with the existing available datasets. For instance, there were two sources of annotations that we tried to use in this study, that were ensGene and RefGene. Based on our observations, ensGene annotation is not only more comprehensive than RefGene but might be more accurate at some level. For example, we run our pipeline for human and dog genomes with dog’s ensGene and refGene annotations, and received six false positive sliding cases with RefGene and only one false positive sliding event with ensGene.

Number of sequenced transcriptomes, especially for model organisms, which we used in this study, increases rapidly. So it is important to change gene annotations using this new data in order to improve its accuracy.

Also there is a possibility that intron sliding might happen only on short evolutionary distances, while we took quite long distances. For understanding this phenomena more carefully there is a huge need in sequenced genomes, transcriptomes, their further accurate annotation and interpretation for closely and distantly related species.

## Conclusions

In this study we were searching for intron sliding events in mammals in order to understand the presence or absence of such events in this group. We did not find any significant evidence of intron sliding in the primate group as well as in mammals, except one dubious case between human and sheep. These results are supported by high quality transcriptomes and the most complete recent genomes annotations. We assume that intron sliding is indeed a very rare evolutionary event, if it exists at all, and each found case needs a lot of supportive data for detection and confirmation.

## Methods

### Data

Mammalian genome pairwise alignments were obtained through FTP site of the UCSC Genome Browser [28, 29] alongside with the corresponding gene structure annotations. Versions of genome assemblies and gene structure annotations for each species used in the analysis are described in Table 1. In case of the cat, rhesus macaque and chimpanzee genomes, we went along with the previous assembly version, as it has more comprehensive annotation than the newest one.

**Table 1:** List of the species used in the study and information of their genome assemblies

Organism name	Common name	Assembly id	Assembly date	UCSC annotation database^a^	Annotation date
*Homo sapiens*	Human	hg38	Dec 2013	knownGene	Oct 2019
*Pan troglodytes*	Chimpanzee	panTro5	May 2016	ensGene	Feb 2019
*Macaca mulatta*	Rhesus macaque	rheMac8	Nov 2015	ensGene	Feb 2019
*Macaca fascicularis*	Crab-eating macaque	macFas5	Jun 2013	ensGene	Feb 2019
*Papio anubis*	Olive baboon	papAnu4	Apr 2017	ensGene	Jun 2019
*Chlorocebus sabaeus*	Green monkey	chlSab2	Mar 2014	ensGene	Feb 2019
*Callithrix jacchus*	Common marmoset	calJac3	Mar 2009	ensGene	Nov 2016
*Mus musculus*	Mouse	mm10	Dec 2011	knownGene [ref]	Oct 2019
*Rattus norvegicus*	Rat	rn6	Jul 2014	ensGene	Feb 2019
*Heterocephalus glaber*	Naked mole-rat	hetGla2	Jan 2012	ensGene	Feb 2019
*Canis lupus familiaris*	Dog	canFam3	Sep 2011	ensGene	Feb 2019
*Felis catus*	Cat	felCat8	Nov 2014	ensGene	Aug 2018
*Bos taurus*	Cow	bosTau9	Apr. 2018	RefGene	Jun 2019
*Ovis aries*	Sheep	oviAri4	Nov 2015	RefGene	Oct 2018
*Sus scrofa*	Pig	susScr11	Feb 2017	ensGene	Feb 2019
*Monodelphis domestica*	Gray short-tailed opossum	monDom5	Oct 2006	ensGene	Feb 2019

^a^ refGene consists of RefSeq gene models in the UCSC, ensGene is a dataset of Ensembl gene predictions, and knownGene contains protein coding genes based on proteins from UniProtKB and their corresponding mRNAs from GenBank

### Search for intron sliding

Using human intron coordinates as reference we were looking for cases when intron coordinates in another organism shifted together over the same distance (but no longer than 5 bp) in their pairwise alignment.

**Fig. 3:**
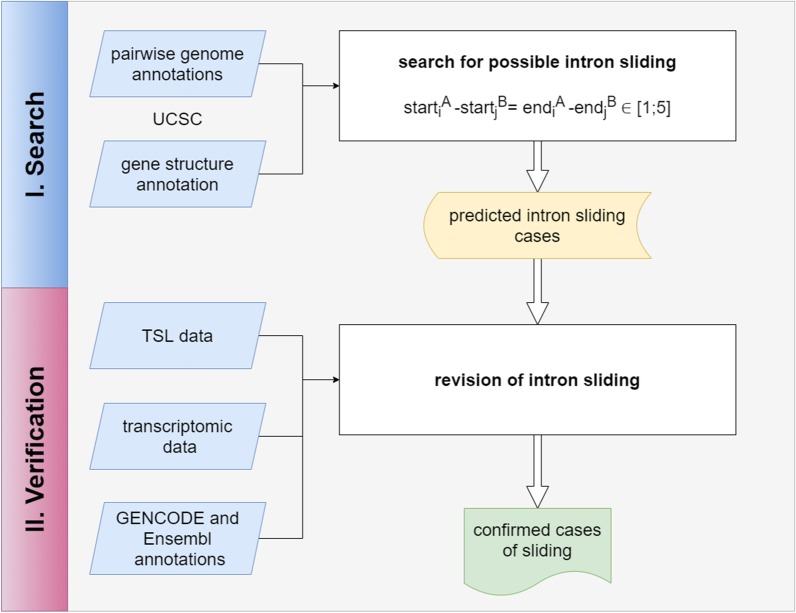
Pipeline of intron sliding identification. start_i_^A^ and end_i_^A^ are coordinates of i-th intron of organism A (human), start_j_^B^ and end_j_^B^ are coordinates of j-th intron of organism B (other mammalian), TSL is Transcript Support Level. We predict the sliding event if the difference between the intron starts are equal to the difference between the intron ends and lies in the interval from 1 to 5 bp inclusive

To identify such cases of potential intron sliding between two genomes we implemented the next pipeline (see Fig. 3). For each gene of each genome we picked its canonical isoform that we defined as the transcript with the biggest number of introns and built two lists of all introns from all canonical isoforms for each genome. Then for each exon–intron border of each intron, we found its alignment coordinates and compared the exon–intron border alignment coordinates of two organisms. As the genome alignment between two organisms de facto is a bunch of alignments between separate conservative genome regions, the exon–intron boundary coordinate could fall out of the alignment. Estimation of evolutionary events on the low conservative regions or difficult to assemble regions is an ambiguous task and such cases were skipped. We saved all cases when alignment coordinates differed by less or equal than 5 bp, and the corresponding exon–intron borders were of the same type (e.g. both of them were starts of the corresponding sequence, or ends, respectively). If both intron coordinates moved on the equal distance in the same direction, we considered it as the potential sliding event.

Pipeline for intron sliding identification was implemented in Python 3 and is available under request.

### Revision of intron sliding cases

All potential sliding events had to be supported by transcriptomic data. We used the Transcript Support Level (TSL) data from GENCODE gene annotations [25] for each human transcript to avoid the predicted transcripts with no experimental support. We kept only cases for which exon–intron boundaries and same intron positions were verified by TSL. We manually checked whether the splice junctions of interest in the left transcripts were supported by transcriptomic data through the UCSC Genome Browser.

## Supplementary Information

**Additional file 1: Table S1.** Putative intron sliding events in mammalian genomes. **Table S2.** Putative intron border shifting over non-equal distances.

## Data Availability

All data analyzed during this study have been previously published at publicly available databases such as GENCODE [25], Ensembl [26] and UCSC [28]. All results obtained in this study are included in this published article and supplementary information. All scripts for analysis are available under request from I.V. Poverennaya (email: ipoverennaya@gmail.com).
