# Supplementary material for: Is there any intron sliding in mammals?
Source: BMC Evol Biol. 2020 Dec 11;20:164. doi: 10.1186/s12862-020-01726-0 (PMC7730772; doi:10.1186/s12862-020-01726-0)
Supplement: Supplementary file 1 — Additional file 1: Table S1. Putative intron sliding events in mammalian genomes. Table S2. Putative intron border shifting over non-equal distances. [file 12862_2020_1726_MOESM1_ESM.docx]

**Table S1. Putative intron sliding events in mammalian genomes.**

This table contains potential intron sliding cases between human and other mammals which were found in the corresponding genome pairwise alignments and their annotations. For each case there is data about corresponding gene, transcript ids, length of sliding and the alignment itself. Intron sequences are written in lowercase letters. The column “TSL flag” contains the TSL category from GENCODE assigned to this particular transcript (“TSL5*” means that there is an alternative transcript with TSL1 and the same intron position). The column “transcript version” shows whether the transcript version from the UCSC annotation used in the analysis has been updated in the Ensembl database. “Transcriptome support” column indicates whether the latest annotation is supported by transcriptome data in the UCSC Genome Browser (if “updated'' positive, then it implies on the updated version). Organism ids are next: hg38 is for human; chlSab2 - green monkey; calJac3 - common marmoset; mm10 - mouse; rn6 - rat; hetGla2 - naked mole-rat; canFam3 - dog; felCat8 - cat; bosTau9 - cow; oviAri4 - sheep; susScr11 - pig; monDom5 - gray shot-tailed opossum.

| **alignment** | | **TSL flag** | **transcript version** | **transcriptome support** |
| --- | --- | --- | --- | --- |
| IGSF5 gene; ENST00000380588.4 (hg38) / ENSCSAT00000006363.1 (chlSab2); sliding 4 bp | | | | |
| hg38 | TCCTCACCAG\|gtagtt...ctgttgccag\|CGGGCTG | TSL1 | - | yes |
| chlSab2 | CCCTTG\|acaggtggtt...ctgttg\|CCAGGAGGCTG | - | - | NA |
| comment: |  |  |  |  |
| PRPF6 gene; ENST00000266079.5 (hg38) / ENSMUST00000136481.7 (mm10); sliding 1 bp | | | | |
| hg38 | TGGCACTGAG\|gtgaggc...cccccag\|GAGCAGCAG | TSL1 | - | yes |
| mm10 | TGGAACTGA\|ggtgaggc...tcccca\|GGAGCAACAG | TSL5 | - | no |
| comment: |  |  |  |  |
| SSPO gene; ENST00000378016.4 (hg38) / ENSMUST00000169350.8 (mm10); sliding 4 bp | | | | |
| hg38 | GCCAGGGTGG\|gtctgg...ccctaggagg\|TGACTTC | TSL5 | - | no |
| mm10 | GTCAGG\|gttggtctgg...c---ag\|GTGGCGATTTC | TSL5 | - | yes |
| comment: |  |  |  |  |
| SPIC gene; ENST00000551346.2 (hg38) / ENSMODT00000003781.2 (monDom5); sliding 4 bp | | | | |
| hg38 | GGAAAAG\|gtacgttgat...ttttag\|GCAGGAAGAA | TSL1 | - | yes |
| monDom5 | GGCAAAGGTGT\|gttgag...atttagggag\|AAAGAA | - | updated | NA |
| comment: | no sliding after update |  |  |  |
| C3 gene; ENST00000245907.11 (hg38) / ENSMODT00000002659.3 (monDom5); sliding 4 bp | | | | |
| hg38 | TTGCACTCAG\|gtgaggcccn...cag\|GCAGTGACAT | TSL1 | - | yes |
| monDom5 | CTGCAGACTGGTGA\|gtgatg...cagggag\|TGACAT | - | updated | yes |
| comment: | no sliding after update supported by 1 mRNA |  |  |  |
| FHL2 gene; ENST00000344213.9 (hg38) / ENSMODT00000038813.1 (monDom5); sliding 4 bp | | | | |
| hg38 | TGACTGCAAG\|nnnnnnn...ccccacag\|GACTTGTC | TSL1 | - | yes |
| monDom5 | TGACTGCAAGGTAT\|nnn...ttctgcaggatc\|TGTC | - | retired | NA |
| comment: |  |  |  |  |
| SWI5 gene; ENST00000418976.1 (hg38) / ENSMODT00000007469.2 (monDom5); sliding 4 bp | | | | |
| hg38 | AAACTAG\|gtgagtagtt...tttcag\|CTGTGATCCG | TSL2 | - | yes |
| monDom5 | AAATTAGGTGA\|gtggtg...ttccagccag\|CATCAG | - | reitred | NA |
| comment: |  |  |  |  |
| PRDX5 gene; ENST00000265462.9 (hg38) / ENSCJAT00000035181.1 (calJac3); sliding 2 bp | | | | |
| hg38 | CTCAAGAG\|gtaaaagt...ccggcag\|GTTCTCCATG | TSL1 | - | yes |
| calJac3 | CTCAAGAGGT\|gaaagt...ctggcagat\|TCTCCATG | - | updated | yes |
| comment: | no sliding after update |  |  |  |
| CATSPER1 gene; ENST00000312106.6 (hg38) / ENSCJAT00000053764.1 (calJac3); sliding 4 bp | | | | |
| hg38 | ACGCAAAA\|gtgagtcttt...tttcag\|CCGGCCGGC | TSL1 | - | yes |
| calJac3 | ACGCAAAAGTGA\|gtcctt...tttcagttag\|CCAGC | - | updated | NA |
| comment: |  |  |  |  |
| OAT gene; ENST00000368845.5 (hg38) / ENSCJAT00000024507.2 (calJac3); sliding 4 bp | | | | |
| hg38 | AGGAAAAG\|gtacgtttca...ttttag\|GTATTTACT | TSL1 | - | yes |
| calJac3 | AGGAAAAGGTAT\|gtttaa...ttttaggcgt\|TTACT | - | updated | yes |
| comment: | no sliding after update |  |  |  |
| ZNF263 gene; ENST00000574674.1 (hg38) / ENSCJAT00000038462.2 (calJac3); sliding 2 bp | | | | |
| hg38 | ACTCACACAG\|gttagta...ctttcag\|TGTGAAGTG | TSL2 | - | yes |
| calJac3 | ACTCACAC\|gggttagtt...ctttc\|AGTGTGAAATG | - | updated | NA |
| comment: |  |  |  |  |
| DNAJA2 gene; ENST00000617000.1 (hg38) / ENSCJAT00000014815.1 (calJac3); sliding 3 bp | | | | |
| hg38 | ATCCACTCAA\|gtgaata...tttccag\|AGTATCTTT | TSL2 | - | yes |
| calJac3 | ATCCACT\|caagtgaata...tttc\|CAGAGTATCATT | - | retired | no |
| comment: | no sliding in new record supported by transcriptome |  |  |  |
| CYP2J2 gene; ENST00000468257.2 (hg38) / ENSCJAT00000031378.2 (calJac3); sliding 1 bp | | | | |
| hg38 | GTTCAGCTG\|gtaggagt...ttttcag\|TTTGTGAAG | TSL3* | - | yes |
| calJac3 | ACTCAGCT\|ggtaggagc...ttttca\|GTTTGTGAAG | - | updated | NA |
| comment: | no sliding after update |  |  |  |
| H2BW2 gene; ENST00000355016.7 (hg38) / ENSCJAT00000062454.1 (calJac3); sliding 1 bp | | | | |
| hg38 | GCCCTCAG\|gtacaccaa...ttccag\|AACTTCATTA | TSL2 | - | yes |
| calJac3 | GTTCTCAGG\|tacaccag...ttccaga\|ACTTCAGTA | - | retired | no |
| comment: |  |  |  |  |
| DUSP9 gene; ENST00000370167.8 (hg38) / ENSCJAT00000022158.1 (calJac3); sliding 2 bp | | | | |
| hg38 | GTTCATTG\|gtgagtcca...atctag\|ATGAGGCCTT | TSL1 | - | yes |
| calJac3 | ATTCATTGGT\|gagtcca...acctagat\|GAAGCCTT | - | updated | NA |
| comment: | no sliding after update |  |  |  |
| C20orf85 gene; ENST00000371168.4 (hg38) / ENSCJAT00000037394.2 (calJac3); sliding 2 bp | | | | |
| hg38 | TACATCAAG\|gtttgaaa...attccag\|GTCTTTCCA | TSL1 | - | yes |
| calJac3 | TACATCAAGGT\|ttgaaa...attccagat\|CCTTCCA | - | updated | yes |
| comment: | no sliding after update |  |  |  |
| NCR2 gene; ENST00000373086.3 (hg38) / ENSCJAT00000008014.1 (calJac3); sliding 3 bp | | | | |
| hg38 | GGCACCAG\|gtgggcggc...ttgcag\|GGGGGACATA | TSL1 | - | yes |
| calJac3 | GACCCCAGGTG\|ggcagc...ttgcagcag\|GTACAAG | - | retired | NA |
| comment: |  |  |  |  |
| ADGB gene; ENST00000367489.2 (hg38) / ENSCJAT00000038187.2 (calJac3); sliding 2 bp | | | | |
| hg38 | GGAAATGCAG\|gtgagtc...ggaacag\|GACTCCTTA | TSL2 | - | yes |
| calJac3 | GGAAATGC\|gggtgagtc...ggaac\|AGGACTCCTTA | - | updated | NA |
| comment: | no sliding after update |  |  |  |
| FBXW12 gene; ENST00000296438.9 (hg38) / ENSCJAT00000005278.1 (calJac3); sliding 3 bp | | | | |
| hg38 | CAACAGACAG\|gtaagc...cattgacag\|CTGCATCT | TSL1 | - | yes |
| calJac3 | TATCACA\|tcggtaagc...cattgg\|CAGGTTTATCT | - | retired | NA |
| comment: |  |  |  |  |
| UBE2G2 gene; ENST00000478200.1 (hg38) / ENSCJAT00000020504.2 (calJac3); sliding 2 bp | | | | |
| hg38 | GAGTACAAAC\|gtgagt...ttttatag\|AATTAACAC | TSL4* | - | yes |
| calJac3 | GAGTACCA\|ttctgagt...ttttat\|AGAATTAACAC | - | updated | yes |
| comment: | no sliding after update |  |  |  |
| SSPO gene; ENST00000378016.4 (hg38) / ENSCAFT00000007296.4 (canFam3); sliding 4 bp | | | | |
| hg38 | GCCAGGGTGG\|gtctgg...cctaggagg\|TGACTTCT | TSL5 | - | no |
| canFam3 | GCCTTG\|gtgggtctgg...tccag\|GAGGTGATTTCT | - | retired | yes |
| comment: | intron position is saved in new record and supported by transcriptome |  |  |  |
| EIF1AX gene; ENST00000379607.10 (hg38) / NM_001145179 (oviAri4); sliding 2 bp | | | | |
| hg38 | AAGAATAAAG\|gtaatgc...ccatcag\|GTAAAGGAG | TSL1 | - | yes |
| oviAri4 | AAGAATAA\|gggtaatgc...ccatt\|AGGTAAAGGAG | - | - | yes |
| comment: | Both sliding and no sliding are supported by numerous mRNAs. |  |  |  |
| Adamts20 gene; ENST00000395541.3 (hg38) / ENSRNOT00000047388.5 (rn6); sliding 4 bp | | | | |
| hg38 | CATGGGG\|atcagtaagc...ttcttg\|TCAGTGCTCC | TSL5 | - | NA |
| rn6 | CTTGGGGAGCA\|gtaagc...tccccatcag\|TGCTCC | - | - | NA |
| comment: | alternative human transcript has the same intron position as rat's |  |  |  |
| SSPO gene; ENST00000378016.4 (hg38) / ENSRNOT00000035906.3 (rn6); sliding 4 bp | | | | |
| hg38 | GCCAGGGTGG\|gtctgg...ccctaggagg\|TGACTTC | TSL5 | - | no |
| rn6 | GTCAGG\|gtgggtctgg...c---ag\|GTGGTGAGTTC | - | - | no |
| comment: | 1 mRNA supports the same acceptor splice sites as human, but different donor splice site. |  |  |  |
| SSPO gene; ENST00000378016.4 (hg38) / ENSSSCT00000045656.1 (susScr11); sliding 4 bp | | | | |
| hg38 | GCCAGGGTGG\|gtctgg...ccctaggagg\|TGACTTC | TSL5 | - | no |
| susScr11 | GCCAGG\|gtgcgtctgg...ctccag\|GAGGTGATTTC | - | - | NA |
| comment: |  |  |  |  |
| TMEM236 gene; ENST00000377495.2 (hg38) / ENSSSCT00000012080.3 (suScr11); sliding 4 bp | | | | |
| hg38 | CAAAAGA\|gtaagtgttc...ttgcag\|GTAGTGAAAA | TSL2 | - | yes |
| susScr11 | CAAAGGAGTAA\|gtattc...ttgcaggtag\|TGAAGA | - | updated | no |
| comment: | intron position is saved in new record, but no transcriptome support |  |  |  |
| ZFYVE26 gene; ENST00000555452.1 (hg38) / ENSSSCT00000002559.3 (suScr11); sliding 5 bp | | | | |
| hg38 | ATCTGCTGAG\|gtaagg...cctttttcag\|ATGTGGG | TSL1 | - | yes |
| susScr11 | ATCAG\|-tgaggtaagg...tattt\|TTCAGATGTGGA | - | - | no |
| comment: |  |  |  |  |
| MICLK/MICALCL genes; ENST00000256186.2 (hg38) / ENSHGLT00000011665.1 (hetGla2); sliding 4 bp | | | | |
| hg38 | AAGGAGG\|gtgagtatg-...ctttag\|AGAGCCAGAA | TSL5 | - | no |
| hetGla2 | AAGGAAGGTGA\|gtgtgg...ttttagagag\|TCAGAA | - | - | NA |
| comment: |  |  |  |  |
| RM30 gene; ENST00000338148.8 (hg38) / ENSHGLT00000009092.1 (hetGla2); sliding 2 bp | | | | |
| hg38 | ATTTGATAAG\|gtttgt...gtcacag\|AATCAAGCCC | TSL1 | - | yes |
| hetGla2 | ATTTGATA\|aggtttgt...gtcac\|AGAATCCAGCCC | - | - | NA |
| comment: |  |  |  |  |
| SEP14 gene; ENST00000388975.4 (hg38) / ENSHGLT00000023201.1 (hetGla2); sliding 1 bp | | | | |
| hg38 | GGAGATACA\|gttaagta...tttaag\|CAAAAAGAAA | TSL2 | - | yes |
| hetGla2 | GAAGATACAG\|ttaagta...tttaagg\|AAAAAGAAA | - | - | NA |
| comment: |  |  |  |  |
| IFT80 gene; ENST00000489004.5 (hg38) / ENSHGLT00000015871.1 (hetGla2); sliding 2 bp | | | | |
| hg38 | AACCAAAGC\|atatcctt...tgacac\|ATCAAGAATT | TSL5* | - | yes |
| hetGla2 | AACCAAGGCAT\|atcctt...cgtcacat\|CAAGAATT | - | - | NA |
| comment: |  |  |  |  |
| SSPO gene; ENST00000378016.4 (hg38) / ENSFCAT00000024510.2 (felCat8); sliding 4 bp | | | | |
| hg38 | GCCAGGGTGG\|gtctgg...cctaggagg\|TGACTTCT | TSL5 | - | no |
| felCat8 | GCCAGG\|gtgggtctgg...tctag\|GAGGTGATTTCT | - | - | NA |
| comment: |  |  |  |  |
| PROX2 gene; ENST00000556489.3 (hg38) / NM_001192679 (bosTau9); sliding 2 bp | | | | |
| hg38 | TTGGTCCAC\|atatcctt...agataa\|ATCCAGGAGG | TSL1 | - | yes |
| bosTau9 | GCGGCCCACAT\|atcctt...ggccaaat\|CCAGGAGG | - | updated | yes |
| comment: | No sliding after update |  |  |  |
| SSPO gene; ENST00000378016.4 (hg38) / NM_174706 (bosTau9); sliding 4 bp | | | | |
| hg38 | GCCAGGGTGG\|gtctgg...cctaggagg\|TGACTTCT | TSL5 | - | no |
| bosTau9 | GCCAGG\|gtgggtgtgg...tccag\|GAGGTGATTTCT | - | - | yes |
| comment: |  |  |  |  |
| TXLNB gene; ENST00000358430.8 (hg38) / NM_001102051 (bosTau9); sliding 4 bp | | | | |
| hg38 | GGATTAG\|gcaagtagtt...tttaag\|GCAAAGAAGC | TSL1 | - | yes |
| bosTau9 | GGATTAGGCAA\|gtagtt...tttaaggcaa\|AGAAGC | - | updated | yes |
| comment: | No sliding after update |  |  |  |

**Table S2. Putative intron border shifting over non-equal distances.**

This table contains cases of the potential intron border shifting over non-equal distances between human and other mammals found in the corresponding genome pairwise alignments and their annotations. For each case there is data about corresponding gene, transcript ids, lengths of both border shifting, and the alignment itself. Intron sequences are written in lowercase letters. The column “TSL flag” contains the TSL category from GENCODE assigned to this particular transcript (“TSL5*” means that there is an alternative transcript with TSL1 and the same intron position). The column “transcript version” shows whether the transcript version from the UCSC annotation used in the analysis has been updated in the Ensembl database. “Transcriptome support” column indicates whether the latest annotation is supported by transcriptome data in the UCSC Genome Browser (if “updated” positive, then it implies on the updated version). Organism ids are next: hg38 is for human; rheMac8 - rhesus macaque; papAnu4 - olive baboon; calJac3 - common marmoset; mm10 - mouse; rn6 - rat; canFam3 - dog; felCat8 - cat; bosTau9 - cow; oviAri4 - sheep; susScr11 - pig.

| **alignment** | | **TSL flag** | **transcript version** | **transcriptome support** |
| --- | --- | --- | --- | --- |
| UBE2J2 gene; ENST00000422076.5 (hg38) / ENSPANT00000033382.1 (papAnu4); shifts (bp) -2 1 | | | | |
| hg38 | TTCTCGAGTG\|gtaaggc...cttccag\|GTTCAAGCG | TSL5 | - | NA |
| papAnu4 | TTCTGGAG\|tggtaaggc...cttccaga\|TTCAAGCA | - | - | NA |
| comment: | - |  |  |  |
| MYO10 gene; ENST00000274203.13 (hg38) / ENSPANT00000005928.2 (papAnu4); shifts (bp) -5 4 | | | | |
| hg38 | ---ATGCCAG\|gtgagtc...ccacag\|CCAGTGGTTC | TSL5* | - | yes |
| papAnu4 | CCCAT\|gccaggtgagtc...ccacagccag\|TGGTTC | - | - | NA |
| comment: | - |  |  |  |
| NXNL2 gene; ENST00000375855.3 (hg38) / ENSPANT00000057785.1 (papAnu4); shifts (bp) -2 1 | | | | |
| hg38 | CCTACCGGCA\|gtgagt...ttttcag\|ACGGAGTCTC | TSL1 | - | yes |
| papAnu4 | CCTACCGG\|cagtgagt...tttcc---\|TGGAGTCTT | - | - | no |
| comment: | - |  |  |  |
| SLAIN2 gene; ENST00000510595.1 (hg38) / ENSMUST00000043711.8 (mm10); shifts (bp) 4 -2 | | | | |
| hg38 | AAAAGAG\|gtaactaca...c----acag\|TACCTTCT | TSL4 | - | NA |
| mm10 | AAAAGAGGTAA\|ctacg...cgcatac\|AGTACCTTCT | TSL5 | - | NA |
| comment: | - |  |  |  |
| TRIM64C/TRIM43B genes; ENST00000617704.1 (hg38) / ENSMUST00000167113.7 (mm10); shifts (bp) -4 -1 | | | | |
| hg38 | TGCAGGGTAC\|gtgatgc...ttgcaag\|AGAAACTTA | TSL5 | - | NA |
| mm10 | AAAATG\|gtaagtgatgt...ttacag\|GAGGAACTTC | TSL1 | - | yes |
| comment: | - |  |  |  |
| CUL2 gene; ENST00000421317.4 (hg38) / ENSCJAT00000063211.1 (calJac3); shifts (bp) -1 -2 | | | | |
| hg38 | TTTGGCTTAG\|gtaggtt...ttgacag\|ATTTCAACA | TSL2 | - | yes |
| calJac3 | TTTGGCCTA\|ggtaggtt...ttgac\|AGATTTCAACA | - | updated | yes |
| comment: | only 1 shifted border after update |  |  |  |
| CBY3 gene; ENST00000376974.5 (hg38) / ENSCJAT00000019377.3 (calJac3); shifts (bp) 4 3 | | | | |
| hg38 | TGGGGATG\|gtatgtacc...tcccag\|CAGCGCCCCC | TSL2 | - | yes |
| calJac3 | TGGGGATGGCAT\|gtacc...tcccagcag\|C-CCCTC | - | retired | no |
| comment: | no transcriptome support for retired record |  |  |  |
| ADAM19 gene; ENST00000517905.1 (hg38) / ENSCJAT00000037162.2 (calJac3); shifts (bp) 4 1 | | | | |
| hg38 | ATGGACAA\|gtaagtggcc...ttggcag\|GAGGAAGT | TSL5 | - | NA |
| calJac3 | ATGGACAAGTAA\|gtggcc...ttggcagg\|AGGAAGT | - | updated | yes |
| comment: | no shifts after update |  |  |  |
| CYTH3 gene; ENST00000396741.3 (hg38) / ENSCJAT00000030067.2 (calJac3); shifts (bp) 4 -2 | | | | |
| hg38 | GTGGCG\|gtgagtgtcg...ttctttcag\|TGCCTGAA | TSL5* | - | yes |
| calJac3 | --GGCGGTGA\|gtgtcg...ttctttc\|AGTTCCTGAA | - | retired | no |
| comment: | no transcriptome support for retired record |  |  |  |
| PER3 gene; ENST00000377532.7 (hg38) / ENSCAFT00000031273.3 (canFam3); shifts (bp) -4 -3 | | | | |
| hg38 | TGAGTAT\|gtaagtgat...tttctag\|CAGTGTGTTA | TSL1 | - | yes |
| canFam3 | TAAATATGGAA\|gtgac...ttcct-gcag\|GGTGTCT | - | updated | NA |
| comment: | 1 shifted border stayed. Another was altered due to the sequence changes |  |  |  |
| IL22RA2 gene; ENST00000296980.7 (hg38)/ ENSCAFT00000000408.3 (canFam3); shifts (bp) -4 -1 | | | | |
| hg38 | GGTGTAGCAG\|gtaagt...atcttag\|GAACTCAGTC | TSL1 | - | yes |
| canFam3 | GCTGTA\|gtaggtaagt...atttta\|GAAACTCAGTC | - | updated | NA |
| comment: | no shifts after update |  |  |  |
| CST1/CST3 genes; ENST00000398402.1 (hg38) / NM_001280713 (oviAri4); shifts (bp) -4 -3 | | | | |
| hg38 | CAGGCAACAG\|gtaggtg...gtgtgcag\|ACCGTTGG | TSL5* | - | yes |
|  | ---GCG\|gcaagcaggtg...atgtc\|CAGGTTGTGTC | - | - | no* |
| comment: | transcriptome supports only 1 shifted border, another one is at the same position as human's |  |  |  |
| DGKH gene; ENST00000611224.1 (hg38) / ENSMMUT00000059128.1 (rheMac8); shifts (bp) -5 4 | | | | |
| hg38 | GACCAAG\|gtagggcgga...tggagacag\|GGTTTCA | TSL2 | - | yes |
| rheMac8 | GACCAAGGTAGG\|gcgga...tggag\|ACGGGGTTTCA | - | retired | no |
| comment: | new version has considerable sequence changes and no shifts. Supported by transcriptome |  |  |  |
| PUDP gene; ENST00000424830.6 (hg38) / ENSMMUT00000024440.3 (rheMac8); shifts (bp) 4 1 | | | | |
| hg38 | CTTCTGG\|gtgggtagt...tctgcag\|GTTATACAGG | TSL3 | - | yes |
| rheMac8 | CTTCTGGGTGG\|gtagt...tctgcaag\|TTATACAGG | - | retired | no |
| comment: | new version has considerable sequence changes and no shifts. Supported by transcriptome |  |  |  |
| DEFB133/Defb49 genes; ENST00000398721.5 (hg38) / ENSRNOT00000060805.1 (rn6);  shifts (bp) -1 1 | | | | |
| hg38 | TGCCACCAGG\|taaaatg...tttaaca\|GTGAAATGT | TSL1 | - | yes |
| rn6 | TGAAGACAG\|gtaaaatg...tttcacag\|TAAAAGCT | - | - | yes* |
| comment: | only 1 mRNA record as transcriptome evidence |  |  |  |
| POU5F1 gene; ENST00000383524.4 (hg38) / ENSSSCT00000001516.4 (susScr11); shifts (bp) -3 | | | | |
| hg38 | CCCGGAGGAG\|gcaagt...aaaatccag\|TCCCAGGA | TSL1 | - | yes |
| susScr11 | CCCCGA\|-gaggcgagt...taaat\|GCAGTCCCAGGA | - | updated | NA |
| comment: | only 1 shifted border after update |  |  |  |
| POU5F1 gene; ENST00000437747.6 (hg38) / ENSSSCT00000001516.4 (susScr11); shifts (bp) -3 -4 | | | | |
| hg38 | CCCGGAGGAG\|gcaagt...aaaatccag\|TCCCAGGA | TSL1 | - | yes |
| susScr11 | CCCCGA\|-gaggcgagt...taaat\|GCAGTCCCAGGA | - | updated | NA |
| comment: | only 1 shifted border after update |  |  |  |
| POU5F1 gene; ENST00000429603.2 (hg38) / ENSSSCT00000001516.4 (susScr11); shifts (bp) -3 -4 | | | | |
| hg38 | CCCGGAGGAG\|gcaagt...aaaatccag\|TCCCAGGA | TSL1 | - | yes |
| susScr11 | CCCCGA\|-gaggcgagt...taaat\|GCAGTCCCAGGA | - | updated | NA |
| comment: | only 1 shifted border after update |  |  |  |
| POU5F1 gene; ENST00000434616.2 (hg38) / ENSSSCT00000001516.4 (susScr11); shifts (bp) -3 -4 | | | | |
| hg38 | CCCGGAGGAG\|gcaagt...aaaatccag\|TCCCAGGA | TSL1 | - | yes |
| susScr11 | CCCCGA\|-gaggcgagt...taaat\|GCAGTCCCAGGA | - | updated | NA |
| comment: | only 1 shifted border after update |  |  |  |
| POU5F1 gene; ENST00000259915.13 (hg38) / ENSSSCT00000001516.4 (susScr11); shifts (bp) -3 -4 | | | | |
| hg38 | CCCGGAGGAG\|gcaagt...aaaatccag\|TCCCAGGA | TSL1 | - | yes |
| susScr11 | CCCCGA\|-gaggcgagt...taaat\|GCAGTCCCAGGA | - | updated | NA |
| comment: | only 1 shifted border after update |  |  |  |
| ACACB gene; ENST00000538526.5 (hg38) / ENSFCAT00000048508.1 (felCat8); shifts (bp) -5 -1 | | | | |
| hg38 | ATGAAAG\|gtaagcccct...catgtag\|GGTGCCTGG | TSL5 | - | NA |
| felCat8 | ATGAAAGGTAAA\|gccac...cacataag\|GTGCCTGG | - | updated | NA |
| comment: | - |  |  |  |
| RGS3 gene; ENST00000374134.7 (hg38) / NM_001077973 (bosTau9); shifts (bp) 3 2 | | | | |
| hg38 | ATGCAAGGAG\|gtagga...gcctccccag\|GTCAACC | TSL1 | - | yes |
| bosTau9 | GTGCAAG\|gaggtaggg...gcc-ccgc\|AGGTAAACC | - | updated | yes |
| comment: | no shifts after update |  |  |  |
